# Annual Address to the Æsculapian Medical Society, Delivered October 30th, 1856

**Published:** 1857-02

**Authors:** H. R. Payne

**Affiliations:** Marshall, Ill.


### THE NORTH-WESTERN

### MEDICAL AND SURGICAL JOURNAL.

(NEW SERIES.)

VOL. VI. FEBRUARY, 1857. NO. 2.

### ORIGINAL COMMUNICATIONS.

ARTICLE I.—*Annual Address to the Æsculapian Medical Society, delivered October 30th,* 1856. By H. R. PAYNE, M.D. of Marshall, Ill.

*Mr. Chairman, Ladies and Gentlemen, and Gentlemen of the Medical Society* — By a constitutional provision of our Society, it is required that some one of its members deliver a public address at each meeting. At the last meeting of the Society I was appointed as speaker for this evening; and, although I distrusted my ability to present anything very interesting or original for your consideration, I felt that a sense of duty at least justified me in making the attempt.

It is my purpose to call your attention to what I consider the true position of Medicine; its claims upon the wants of mankind; and to refer you to some of the causes which have, in a great degree, retarded the progress of our science.

The practice of medicine, as has been very appropriately said, has its foundation in nature. The individual prostrated by disease, burning with fever, and suffering from the most intense pain, cries for relief. And where, I would ask, if not in nature—in the great kingdom of nature—could we find those means which, when properly applied, have the power of relieving suffering, controlling disease, and prolonging life? That medicine possesses this power when judiciously applied, there is no competent observer of our profession who will deny. We do not wish to be understood as saying, that medicine is always curative. The experience of the past does not warrant us in the conclusion that medicine is invariably certain. We would be false to medical history, and false to the experience of our own times should we assert otherwise. There are many diseases with which we have to contend in which medicine exerts but a feeble, a palliative effect. There are many persons in the world, however, who believe that because medicine is not invariably certain; because it fails sometimes in arresting disease, that it is fraught with evil, and confers no benefit upon mankind: but such persons take a very limited view of the subject. It must be recollected that the laws or principles which govern animate are far more complex than those which govern inanimate matter. If we discard medicine because we are sometimes disappointed in it; because it fails, if you please, in many instances in arresting disease: with the same propriety might we discard such for example as the art of government, political economy, &c. on account of their imperfections. In fact we might discard all republican institutions on the same principle, because there is not unlimited freedom, or because forsooth it did not come up to our standard of right.

This spirit of skepticism in the efficacy of medicine has always existed and probably ever will; and I am sorry to say it exists not only outside of the profession, but we frequently hear physicians themselves expressing their entire disbelief in the power of medicine as a curative agent.

Gentlemen! if a man is satisfied in his own mind that medicine is of no avail; that it has no power in controlling disease; that it confers no substantial benefit on his race: I ask, would it not be better for him, as a conscientious man, to renounce his profession and seek some other as a means of livelihood?

As I have already stated, we do not claim for medicine invariable certainty. The important part that nature plays in the cure of disease should never be lost sight of by the individual who practices the healing art. In fact, without recognizing this principle, we are liable to be led into error, and in many cases may do much serious harm. That medicine has the power, if applied judiciously, of assisting nature in the cure of disease, and in many cases of saving life, when the energies of the system are powerless, has long since been established by the most competent observers of our art. If, then, it can be made clear, that, by the application of a certain remedy in a certain disease, is followed by recovery, when the disease, unassisted by art, would have terminated fatally, it ought to be acknowledged by the most skeptical that so far at least it deserves the praises of mankind.

Perhaps this cannot be established more clearly than by referring to a disease fatal in its character, and which is of frequent occurrence in some parts of our Western country. I refer to Pernicious Fever, or what is more commonly known as Congestive Chill. This disease terminates fatally frequently in the second chill, and almost invariably in the third. When the patient is seized with one chill, it is as certain to return as the chill of an intermitting fever will be followed by another paroxysm. Here, then, is a disease which, if left to itself or to nature, almost invariably terminates fatally. But, on the other hand, if the case can be seen at the proper time, and the proper remedies are applied, the great majority of these cases can be saved. In the early settlement of our country this disease prevailed and was little understood, and hence was fatal in almost every instance. Now, however, owing to the improvements in medicine, we seldom hear of a fatal case, without the patient has died in the first chill, or no treatment has been pursued.

Again, Bilious Fever is a disease which, if unassisted by art, nature will in some cases eventually throw off: but this is not invariably the case. Let us see: when the case is left to nature a double office is performed. The patient lies in the most intense suffering for a number of days before the disease yields. Nature, having a greater work to accomplish, requires a longer time to do her office, and, unfortunately, in many instances, is incompetent for the task, and the patient passes into a low or typhoid state, which in many cases terminates fatally. This is no sketch of the imagination: I doubt whether

4*

there be a physician present, of any great experience, but what can refer to such cases; cases where the administration of the proper remedies were neglected, and the patient relapsed into the condition I have just described. Even the simplest form of bilious fever (I refer to intermitting fever), if neglected or allowed to pursue its own course, will in some instances terminate in congestion, that much dreaded disease we have just described. If not, it invariably debilitates the system, and makes it an easy prey to other diseases.

Medicine, then, is not only curative, but, when it fails in this, it assists the efforts of nature in expelling the disease. It not only does this, but it has the power of stopping, temporarily, the extreme pain which frequently is the chief element in the disease. If practical medicine, then, can claim these triumphs, has it not conferred a great benefit on our race? and should it not excite us, who are its humble followers, to greater diligence, with the view of finding out other means which will have the power of controlling diseases which are now imperfectly understood. The spirit of medical inquiry is still progressing, and we are encouraged in the hope that the time is not far distant when we will possess the power to control those diseases now regarded as incurable. This hope is not unreasonable. But a few years ago, physicians regarded *Pulmonary Consumption* as incurable; now, however, by recent developments and discoveries in medical science, it is established that many cases of this disease may be cured; and the impression is growing in the profession that we will soon command the remedy that will stay its progress.

In no department of our science has so many valuable discoveries been made recently as in *Diagnosis*, or the mode of distinguishing disease. But a few years ago, owing to imperfect diagnosis, many diseases were involved in much obscurity: such was emphatically the case with the diseases of the chest. Now that this difficulty is in a great measure obviated, we can apply our remedies with greater certainty of success. The name of Laennec will be ever held in grateful remembrance as one of the benefactors of his race. Marshall Hall and other discoverers will not be forgotten for unfolding to us many valuable facts relating to the mysterious workings of the nervous system.

But the object of our science is not only to understand the nature of disease; not only to recognize it, and point out the means by which it may be arrested: but it seeks a higher purpose. The science of medicine embraces everything within its scope which relates to the causes so prolific of disease, and the means by which those causes may be counteracted. The finding out of these causes, with the view of preventing disease, has been a subject of investigation which has taxed and in many instances baffled the ingenuity of many of our most learned men. It is true that there are many of these causes so inscrutable that they will probably never be found out; such may be said to be the case with Asiatic Cholera, a disease truly frightful in its character, and one which has carried away its tens of thousands. Physicians have sought, labored, and experimented in vain, and their labors have been attended with no satisfactory results. It is true, that theory after theory has been proposed, but, like all speculations, has been of no avail; because the principle has never been shown or demonstrated as a fact. But, although the cause may forever elude our search, physicians, by their observation and experiments, have found out the means by which it is disarmed of much of its terror, and that is by a strict adherence to sanitary regulations. It is true, it sometimes occurs in places where the greatest precautions have been observed; but these are the exceptions to the rule. It is well known to almost every one at all acquainted with the spread of this disease, that, when it breaks out in cities or towns, its most usual locality is in those places most noted for filth and want of personal cleanliness. It has been shown by observation, that the cause of this malignant disease not only selects such places, but that *these* places give intensity to its action. This applies not only to epidemic cholera, but to all other diseases of an epidemic or contagious nature.

The progress of our science up to the last century was slow. It is true it made advances in anatomy, physiology, and chemistry, but practical medicine did not advance with the same ratio. This must be attributed to the fact, that the leading men in the profession were governed in their practice by their theories. They did not feel like yielding obedience to the labor of collecting and examining facts. They preferred to take up a theory of their own formation, not founded in nature, and deduce from it certain principles to guide them in the treatment of the diseases they had to contend with. But, like all speculation, they soon died away. Thus, for ages, has theory after theory successively risen, and before the generation in which it was promulgated had passed away a new one was reared in its stead. Where, I would ask, are the theories of Galen and of Paracelsus, of antiquity? And to come down to more modern times, I would ask, Where can be found a follower of the fine-spun theories of Brown, of Broussais, and of our own countryman, Dr. Rush! And it is unfortunate for the progress of our profession that we have men, even in the present day, who prefer following a theory than they do to follow the only sure way to advance the progress of medicine, and that is *observation* and *experience.* I do not wish to be understood as saying that experience alone qualifies the man. To arrive at any degree of eminence, he must read, reflect, and, above all, observe. A man may practice medicine for years, and without he has a capacity for observation he can never apply his experience to advantage. His practice will be a routine; and although he may have been engaged in it for forty years, his knowledge from his experience will be but little better than the day he first embarked in the laborious duties of his profession. I am induced to make these remarks from a mistaken notion of many persons, that experience alone qualifies or makes perfect the physician. If, then, observation and experience be the great source from whence we are to derive our knowledge in the treatment of disease, we can here see one great obstacle to the advance of medicine. It is true, it cannot, like the physical sciences, arrive at the same degree of certainty; but it can arrive at this in a great degree; in other words, it can approximate a certainty. And to even approximate a certainty, the most patient investigation and observation is necessary. To quote the remarks of an able writer:—'An age of close observation may be required to determine *that*, which *experiment*, were in our power, would determine in a day.'

But there are other causes which retard the progress of legitimate medicine. It would be reasonable to suppose, as new facts in science, and new wonders in the material world are laid bare, that, in like proportion, would superstition and the various delusions disappear. But such is not necessarily the case. We, of this enlightened generation, are astonished that a man of the character of Lord Bacon; a man distinguished in position, in wisdom, and in philosophy; possessing a name that will be handed down with pride from one generation to another; should have so far degraded himself as to believe in the existence of witches, enchanters, &c. Bacon even believed, it is said, in sympathetic cures. He believed, for example, that the rubbing of warts with a piece of fat bacon, and then hanging it up to the sun, to go through a drying process, would cause them to entirely disappear. It is said that James the Sixth believed that witches infested the country, and he maintained the necessity of punishing them. At one time the opinion was prevalent that scrofula, or king's evil as sometimes called, could only be cured by the royal touch—and it required the work of several successive generations before this superstition could be removed. Even in the early settlement of our own country the supposition prevailed that witches infested the country; and it is well known to you that lives were even sacrificed as the result of this superstitious belief. And, to come down to our own times, how many persons are there living who believe that certain persons have the power of removing a tumor by the touch, or of stoppiug a hemorrhage by a charm. It is true, however, that the most of these superstitious opinions have disappeared, and although confined to a few individuals it does not pervade generally all classes as formerly. But, although science has done much to dispel them, unfortunately for our generation, we have a new train of delusions which, within a few years, have sprung into existence, and number among their supporters men of intelligence and morality. We do not seem to have profited much from the experience of the past. If our forefathers believed in witches, enchanters, &c. we have substituted a more refined set of delusions, such as *Table-Turning*, *Clairvoyance*, *Spirit-Rappings*, *&c.*; which, although more refined, and probably compatible with the spirit of the age, are equally as delusive and absurd.

One of the most extraordinary medical delusions; one that has obtained among the gay and intellectual, and spread its wings, and extended its principles from one portion of the country to the other, is *Homœopathy*, which, although recognized as a system or doctrine of medicine, hardly deserves the name. This system, like all other systems not based upon facts, cannot stand the test of time. The history of all other systems of medicine teaches us, that, however plausible and captivating it may at first be, if it is constructed upon false premises, or, in other words, not based upon positive facts, it will soon die out. The principle upon which this system is founded, is, *Similia Similibus Curantur:* that is, what will produce a disease, will cure it. To use Hahnemann's own language (who was the founder):—'To cure, in a mild, prompt, and durable manner, it is necessary in each case to choose a medicine that will excite an affection similar to that against which it is employed.' If, for example, an individual is bitten by a bug, he must drink *Bug Tea* of a certain dilution to stop the inflammation. If he is bitten by a venomous reptile, he must drink the venom of that reptile as an antidote. If he be laboring under an inflammatory fever, he must take spirituous wines to cure the fever. Even in inflammation of the brain, the founder of this system contended must be treated Homœopathically, by administering wine. This principle, although in the main incorrect, you will see is attended with great danger, if the remedy is used in sufficient portions to produce any effect. But, even admitting this principle to be correct, what virtue can be attached to the infinitesimal doses they recommend. If any candid person will give the subject one moment's serious consideration, they will see that the doses recommended in disease will have no more weight, in fact ten thousand times less, than a feather in a whirlwind. A Homœopathic pill, containing the decillionth of a grain of pulsatilla, or belladonna, or mercury, is prescribed for the cure of a certain disease. The remedies are prescribed with great care, and the directions given with great exactness; they are continued from day to day; and, finally, after the lapse of days, perhaps of weeks, the disease yields, and the patient recovers to the great satisfaction of the doctor, and with the confident belief on the part of the friends that the remedy obtained the mastery over the disease; when, perhaps, to the knowledge of the medical attendant, it had no agency whatever in the cure. The remedy was given, and the patient recovered; hence, according to the mode of reasoning of many persons, it cures the disease. Whenever the people can learn the great part that nature plays in the cure of disease, and that in many instances, without the assistance of art, she has the power of throwing off disease—*then*, and not until then, will they see the folly of this imaginative and absurd system of practice.

Homœopathy ranks among its followers men of science and fame; but it does not follow, that because a doctrine is enthusiastically embraced by men of a certain stamp of intelligence, that the doctrine is right. I believe that the majority of men who are busy in promulgating this absurd creed; those that are the most loud in disseminating its principles, have but little confidence in it themselves. They are well aware that nature is often sufficient to contend with the disease; but they are too base to make this acknowledgment, because they know that here rests the secret of their success, and this once understood would remove the last vestige of its pretensions. But intelligent Homœpathists do not carry out their principle when they encounter a formidable malady, or one really requiring the interference of art—that is, 'that the smallest possible dose is the proper dose.' There are cases on record where these same men have been found prescribing the same remedies recommended by regular physicians, when they *knew* that medicine was really required. It is well known that we possess many powerful remedies, hence, to carry out the deception, these powerful remedies are used in small doses to produce the desired effect. If, for example, they wish to give a mercurial, as a substitute for calomel, they give *corrosive sublimate*, because it is powerful and can be given in small doses. If it is necessary to prescribe a tonic, they give a small dose of *arsenic*, &c. I would ask any candid man if these developments do not give the contradiction to their whole system of practice? But the great error in Homœopathy arises from the fact, that they attribute results from doses of medicine which are so small as to be incapable of producing any effect whatever. To give you some idea of their minuteness, I would ask: What would be the effect of a drachm of alcohol dissolved in a cistern containing a hundred hogsheads of water, and given in ten drop doses every hour? This is a question which you can all appreciate. Would it be capable of producing any sensible effect on the organism? Would it have any effect of assisting the operations of nature, if the system was sinking from some exhaustive disease? But this illustration falls far short of giving a correct idea of it. Hear what Dr. Simpson, of Edinburgh, a star of the first magnitude in our profession, has to say—a man who is entirely reliable, and has done more to expose the follies of this system than any man living. He says:—'Soon after the promulgation of Hahnemann's doctrines, it was suggested, that "If the decillionth part of a grain has any efficacy, an ounce of medicine (Epsom salts) thrown into the Lake of Geneva would be sufficient to physic all the Calvinists of Switzerland." But more careful systematic calculations have shown that this is stopping infinitely short of the truth; and that the thirtieth Homœopathic dilution, recommended as we have seen by Hahnemann in all cases, is in such a parallel enormously understated instead of overstated. In fact the tenth solution would alone require for its proper solution a body of water five hundred times greater than the bulk of the Lake of Geneva, or a sea somewhat larger than the Gulf of Venice. To make the eleventh solution, a quantity of water greater than the Mediterranean Sea or German Ocean would be necessary. The twelfth solution could scarcely be accomplished in a sea extending over the whole surface of the earth, and five hundred fathoms in depth. And if the whole solar system were buried in an ocean, extending in depth from the Sun to Neptune, it would not form a sufficient fluid medium for adequately dissolving to the thirtieth dilution—a common dose of any of the common medicines of the Homœopathists.' 'Yet the Homœopathists allege that a few drops or sips of the proper medicine, properly dissolved in such enormous medicated seas and oceans, infallibly acts and cures; and that each drop would be of "terrific potency" if the drug were duly mixed.'

This, although it may require a stretch of the imagination, is correct, as it is based upon the most accurate mathematical calculation.

*Hydropathy* is another system of medicine which, although more consistent, frequently goes hand in hand with Homœopathy. It is a system which pretends to combat all disease by the use of water. Now water is a valuable agent, because it is essential to the existence of all beings, whether of animal or vegetable origin. It is also valuable as a medicinal agent; but its use in this respect is not a discovery of recent origin. Years before this system of would-be-reform sprung into existence it was used in the treatment of disease, particularly that of a chronic nature. But to contend that this agent is the remedy to meet all forms of disease, is to my mind just as absurd as to say that it possesses no virtue whatever. Water produces different effects at different temperatures. We have the *hot* bath, the *warm* bath, the *tepid* bath, and the *cold* bath. One, for example, is decidedly stimulant, and frequently depletes rapidly. Another is relaxing. A third is intended more to promote insensible perspiration; whilst the last or cold bath is used more for its tonic and sedative effect.

Now the advocates of this system would try to make the world believe that, whilst the application of water, in its different forms, is the remedy applicable to all kinds of disease, it possesses the advantage of being harmless in its effects. We deny the proposition, that it is applicable for all forms of disease, because it is not sustained by facts, but based upon the weakest of hypothetical reasoning.

We deny, in the second place, that it is harmless in its effects; and for the very reason that it is one of the most powerful agents we possess. And even in cases where it is used with judgment, it is not free from pernicious effects.

A man may be *frozen*, or he may be *steamed* to death—these are familiar illustrations.

Take, for example, cold water. In all cases when it is used it must be followed by reaction, or you run the risk of inflammation. A man, for example, is exposed to a rain; he becomes drenched as it were with the element. He becomes chilled, the blood on the surface is driven to the internal organs, and if reaction is not soon established, it is liable to be followed by serious results, as congestion and inflammation of the lungs, &c.

Again, let an individual be exposed to cold whilst his system is perspiring or relaxed, Will not the effect be to produce serious disease, and in many instances death?

As we have said before, water, if judiciously used in certain diseases, is a valuable agent; but we denounce any practice or doctrine that would build upon it any *exclusive system*, as it is in many cases injurious; and in many other cases when it does not do positive harm, it prevents the use of other means necessary to the recovery of the case.

The *Thompsonian*, or *Steam* system as it is more commonly known, prevailed extensively over our country a few years ago, and threatened at one time to swallow up all other systems. It still prevails in some localities; but it is fast waning, and, like the doctrines we have just referred to, based upon hypothetical reasoning, will soon pass away, and be known and regarded by another generation as one of the greatest follies of our times.

The theory of *Thompson*, who was the founder of this system, was, that the human body is composed of four elements—*Earth*, *Water*, *Air*, and *Fire*. The *solid* parts of the body he considered were composed of the earth and water, and the *fluids* of air and fire. He pretended to make the discovery that heat was life, and that cold was death; hence, all diseased action consisted in a loss of heat, and depended upon obstructed perspiration as the exciting cause. From these general principles he deduces, that all disease, consisting of loss of heat and obstruction of perspiration, required a remedy that would remove the obstruction and restore the loss of heat.

The remedies which seemed to him most effectual in fulfilling these indications, were, *Lobelia* to remove obstruction, as by vomiting; and *Cayenne Pepper*, and, if needs be, *Steam*, to restore the loss of heat.

Any attempt on my part to disprove this position is unnecessary, as the knowledge that it was founded upon hypothesis is the best argument against it—besides, it has not stood the test of experience. That Lobelia, that Cayenne Pepper, or Number Six, or Steam, are beneficial in the treatment of certain diseases, we do not pretend to deny. But to say that they are the remedies required in the treatment of *all* diseases is just as inconsistent with reason, with philosophy, and with experience, as it is in saying that Homœopathy or Hydropathy are adapted to the cure of all diseases.

*Eclecticism* is another name for a new sect which has sprung into existence of late. It is what I would designate as a reformed modification of Thompsonianism. I object to the name *Eclectic* because it implies that they select from all sources whatever is valuable. I object to the system because it is exclusive. They profess to make use of no other remedies but those found in the *vegetable* kingdom. Yet, in the face of this declaration, they call themselves Eclectic—for the sake of clamor, and to please the prejudices of a few people, they denounce the use of calomel and the necessity of the lancet. It is not my purpose here to defend the use of calomel. I am willing to say in candor that it is a remedy which has been abused; that it not only *has been*, but *still is used in many cases where it is not required.* It *has* been the cause of much injury and suffering on account of its constitutional effects; but this is no argument against the necessity of the remedy when judiciously used—and the same may be said of the lancet. Bleeding *has* been too common; but certainly there is no Eclecticism in saying that there are *no cases* requiring its use. The intelligent members of this system know the value of these remedies, and in many cases when they suppose them to be applicable resort to them.

I object to this system, as I have before stated, because it is exclusive. The system of medicine that we advocate is not exclusive. If it has in times past, it is not so much now swayed by speculative or medical doctrines. It selects from the great kingdom of nature *all* of those means, whether of animal, vegetable, or mineral origin, which have been shown by close observation and experience to be applicable to the cure of disease.

But we should not be too severe in our censure of these different doctrines, because they number among them men honest and conscientious; men who, although deluded, are their humble votaries. We should not loose sight of the fact, too, that among the followers of our own profession we have men who have reared up for themselves false theories which have unfortunately in many instances swayed the minds and governed the practice of thousands of those whom we are proud to call our own. These, then, are some of the causes which have in a great degree retarded the progress of legitimate medicine.

Gentlemen, in spite of the difficulties that beset our science, we should not be disheartened. We should recollect that the accumulated experience of the past has not been in vain; the growth of our science has been steady, and in no period of its history has it made such advances as within the last century. We acknowledge it to be imperfect; but it must be recollected that imperfection accompanies all science and art — in fact, without it we could not progress. But, if judging from the past be any index to the future, we will be at least justified in saying that our science is yet destined to a much higher state of perfection. Is it not our duty, then, as the followers of this noble profession, which has for its object 'The relief of the sufferings of humanity,' to renew our vow that we will do all we can to advance our profession in its career of usefulness, prosperity, and glory.

